# The last step to achieve barrier damage control

**DOI:** 10.3389/fimmu.2024.1354556

**Published:** 2024-02-13

**Authors:** Ilaria Baglivo, Stefania Colantuono, Arianna Lumaca, Alfredo Papa, Antonio Gasbarrini, Cristiano Caruso

**Affiliations:** ^1^ Centro Malattie Apparato Digerente (CEMAD) Digestive Disease Center, Fondazione Policlinico Universitario “A. Gemelli” Istituto di Ricovero e Cura a Carattere Scientifico (IRCCS), Università Cattolica del Sacro Cuore, Roma, Italy; ^2^ Unità Operativa Semplice Dipartimentale Day Hospital (UOSD DH) Medicina Interna e Malattie dell’ApparatoDigerente, Fondazione Policlinico Universitario “A. Gemelli” Istituto di Ricovero e Cura a Carattere Scientifico (IRCCS), Università Cattolica del Sacro Cuore, Roma, Italy; ^3^ Unità Operativa Semplice Dipartimentale (UOSD) di Allergologia, Ospedale Maria Santissima (SS) Dello Splendore, Teramo, Italy

**Keywords:** type 2 inflammation, immune system, non-T2 inflammation, epithelial barrier damage, alarmins, eosinophils, tezepelumab

## Abstract

Heterogeneity characterises inflammatory diseases and different phenotypes and endotypes have been identified. Both innate and adaptive immunity contribute to the immunopathological mechanism of these diseases and barrier damage plays a prominent role triggering type 2 inflammation through the alarmins system, such as anti-Thymic Stromal Lymphopoietin (TSLP). Treatment with anti-TSLP monoclonal antibodies showed efficacy in severe asthma and clinical trials for other eosinophilic diseases are ongoing. The aim of this perspective review is to analyse current advances and future applications of TSLP inhibition to control barrier damage.

## 1 Introduction: epithelial damage as a common beginning of allergic eosinophilic diseases

Human epithelium is the border between the external environment and our tissues with the role to maintain homeostasis defending from pathogens, pollutants, toxins, allergens and other external agents. This defence is provided not only by the physical barrier given by Epithelial Cells (ECs) tightness, but also through antimicrobial peptides production, mucociliary clearance, mucus production, permeability regulation (1). When damaged, pathogens, allergens and other irritant agents can penetrate the epithelium and, recognized by the Pattern Recognition Receptors, can induce innate and adaptive immune responses (1). The “barrier hypothesis”, formulated in 2017 by Pothoven and Schleimer, formulates that the epithelial damage chronologically anticipates allergic sensitization and the development of eosinophilic inflammation in many inflammatory diseases. Actually, a defective epithelial barrier has been found in many conditions such as atopic dermatitis, asthma, allergic rhinitis, chronic rhinosinusitis, eosinophilic esophagitis, coeliac disease and inflammatory bowel diseases (2). Herein, we give a general perspective on how this barrier hypothesis enriched our understanding about the pathogenesis of Type 2 (T2) diseases and how it could be exploited for their treatment.

### 1.1 Barrier damage in asthma phenotypes

Asthma is a chronic inflammatory airway disease clinically characterised by reversible airflow obstruction, bronchial hyperresponsiveness and respiratory symptoms (3). Although the allergic and eosinophilic represent the most frequent phenotypes, T2-low or non-T2 mediated asthma is also reported (4, 5).

Recent evidence showed that all asthma phenotypes share a loss of cohesion between the airways’ ECs with an increased permeability probably due to inflammation and to allergens-derived enzymes from House Dust Mites (HDM) or fungi capable of cleaving intercellular junctions worsening barrier damage (6).

ECs have gained increasing clinical importance as awareness about their role in initiating the inflammatory response raised (6). Nowadays, we know that ECs are not a mere physical barrier but cells capable of generating the immunological responses to external triggers. Lung ECs expresses different pattern recognition receptors as Toll-Like Receptors (TLRs), Nucleotide-binding Oligomerization Domain (NOD)-like receptors, protease activated receptors, C-type Lectin Receptors (CLRs), purinergic receptors, Retinoic acid-Inducible Gene (RIG)-I-Like Receptors (RLRs) which allow them to produce chemokines and cytokines in response to different external triggers (6). In turn, these cytokines activate many immune cells involved in asthma, such as Dendritic Cells (DCs), T helper 2 (Th2) cells, mast cells, type 2 Innate Lymphoid Cells (ILC2s), eosinophils, basophils that concur in augmenting and maintaining inflammation through the subsequent damage of the epithelial barrier (6).

Currently the best-known ECs’ cytokines are TSLP, IL-33, and IL-25. These ECs signals are rapidly released in response to various stimuli like air pollutants, bacteria, viruses and allergens such as HDM, Aspergillus fumigatus, and the cat protein Fel d1 (6). Higher concentrations of IL-33 and TSLP, but not IL-25, were shown in asthmatic patients independently of phenotype with levels inversely correlating with lung function (7). Indeed, even if their increase is much more related to allergic and eosinophilic asthma, there is evidence suggesting they can be involved even in non-allergic non-eosinophilic phenotypes. The cells source of TSLP and IL-33 have distinct cellular profiles compared to that producing IL-25, this could explain the discordant expression of alarmins, depending on the clinical context and the airway inflammatory cells sampled (8–10). *In vitro* findings indicate that TSLP and TLR3 ligands promote the differentiation of naive CD4+ T cells into T helper 17 (Th17)-cell via DCs activation and IL-23 production (7). Moreover, *in vivo* studies found out that neutrophils are a source of TSLP in bronchial biopsy tissue and, consistently with this, TSLP expression in bronchoalveolar lavages has been reported to correlate with neutrophilic inflammation (6, 7).

Numerous evidences demonstrated that some chemicals induce TSLP production and may potentially augment T2 allergic responses. In murine allergic asthma model, repeated intranasal exposure to Cigarette Smoke Extract (CSE) induced TSLP production in bronchial epithelial cells and the administration of an anti-TSLP antibody to the same model attenuated CSE-enhanced leukocyte infiltration (11).

CSE induced TSLP production *in vivo* via oxidative stress and Tumour Necrosis Factor (TNF)-a receptor 1 activation. Oxidative stress also induced the production of oxidized lipids, which triggered TSLP production via stimulating TLR4 (12). CSE was shown to increase the expression of TSLP and TSLP-Receptor mRNA in human Airway Smooth Muscle Cells (ASMCs), and this response was not attributed to nicotine or Reactive Oxygen Species (ROS) (13).

### 1.2 Barrier damage in atopic dermatitis

Atopic Dermatitis (AD) is the most common chronic inflammatory skin disease affecting infants, children or that can develop in adolescents and adults as well. Pathophysiology of AD is multifactorial and association with comorbidities as food allergy, asthma and allergic rhinitis is common (14).

Although the underlying mechanisms are not fully understood, evidence suggests that AD is an inflammatory skin disease that releases systemic components able to progress to the lungs if left untreated (level shift) (15). AD is characterised by an interaction between impaired epidermal barrier function, skin inflammation, and skin microbiota dysbiosis. There is undoubtedly a relationship between AD genetic susceptibility, microbiota-related epigenetics, and how barrier restoration and microbiota manipulations affect AD (16).

A marked difference in skin microbiota biodiversity has been found in some studies between patients with AD and those with non-atopic dermatitis (17), as already described in asthma (18).

Scientific attention to the barrier mechanism is increasing and it is well described in several recent articles (19). A recent study described that skin-based impedance spectroscopy may offer an *in vivo* technique for assessing the integrity of all components of the epithelial barrier, also valuable for the assessment of AD severity, progression and therapy efficacy (20). Latest findings showed that TSLP induces the immune responses via activation of DCs and mast cells; therefore, TSLP is considered a key molecule in AD pathophysiology (21). Serum levels of TSLP in adults and children with AD are significantly higher than those in healthy people. In addition, TSLP gene polymorphisms were demonstrated closely related with an increased risk of developing AD and disease progression (22).

### 1.3 Barrier damage in eosinophilic esophagitis

The epithelium of the esophagus protects the deepest mucous and submucous layers from triggers like environmental toxins and allergens. Maintenance of the normal esophageal barrier depends on the correct balance of epithelial, narrow differentiation proteins junctions, adherens junctions and desmosomes (23). Genetic predisposition and environmental factors contribute to the barrier damage balance (24). In Eosinophilic Esophagitis (EoE), allergens cause damage inducing defective esophageal barrier. Basal cell hyperplasia and dilated intercellular spaces have been described in EoE epithelial cells as hallmarks of barrier damage and an important feature of disease (25). Following exposure to food allergens and proteases, esophageal epithelium secretes TSLP and IL-33 inducing Th2 cells to release IL-4, IL-5, and IL-13 that affect: proteins such as the Desmosome Desmoglein-1 (DSG1), epithelial differentiation proteins filaggrin and involucrin, eosinophils, mast cells and basophils infiltration (25–28).

A dysregulation of the protease and antiprotease system is also present (29). A dysfunctional barrier predisposes to a sensitization of food allergenic proteases and other triggers.

A dysregulated neural control of the esophagus is present and directly related to dysphagia in these patients. Nociceptive sensitization within the esophageal mucosa, resulting from inflammation, leads to motor pain, dysfunction and possibly tissue remodelling as in other respiratory and skin diseases, remodelling is mainly inflammation-driven in EoE (30, 31).

### 1.4 Barrier damage in rhinitis and chronic rhinosinusitis

Chronic Rhinosinusitis (CRS) is an inflammatory condition of nasal mucosa divided into CRS with and without Nasal Polyps (NP) (32). The nasal mucosa is the first site for inhaled antigens exposure. Nasal epithelium maintains the immunological barrier and is a metabolically active organ able to produce various protective proteins and secretory IgA with a crucial role in contrasting allergens, microbes and other noxious substances penetration (33). The integrity of the nasal epithelial barrier is essential for ensuring its protective function. The epithelial barrier damage is a feature of the disease and contributes to NP development (34). Epithelial tight junctions are altered in NP: a down-regulation of the expression of ZO-1, E-cadherin, and occludin has been observed (35). Moreover, the epithelium repairment is altered in patients with NP (36).

Examination of histological samples from nasal mucosa epithelium or nasal cytology allows the evaluation of the barrier damage directly or indirectly (37). In a cytological context, the Hyperchromatic Supranuclear Stria (SNS) has been reported as a specific marker for the anatomic and functional integrity of ciliated cells. The absence of the SNS is considered a useful prognostic sign of loss of mucosal barrier integrity in nasal disorders (38).

The nasal microbiome also contributes to the functional sinonasal barrier. In CRS patients, the nasal microbiome appears heterogeneous, with a decrease in bacterial diversity, resulting in dysbiosis. Dysbiosis favours the relative abundance and the chronic colonisation of S. aureus. Furthermore, an increased exposure to antibiotics and steroids has been associated with a lower total bacterial biodiversity (39). The prevalence of bacterial biofilms in the paranasal sinuses in CRS has been reported in about 42–80% of patients, with a higher prevalence in Chronic Rhinosinusitis with Nasal Polyps (CRSwNP) (40). The most frequently detected organisms in CRS biofilms are S. aureus, Haemophilus influenzae, and Pseudomonas aeruginosa (41). It has been shown that S. aureus directly damages airway epithelial cells together with their repair mechanisms regardless of enterotoxins production (42). S. Aureus can be found under the epithelial surface in nasal mucosa, particularly in patients with NP (43). Damaged epithelial cells favour IL-25, IL- 33 and TSLP releasing that activate ILC2-mediated eosinophilic inflammation. In addition, the staphylococcal toxins induce a massive activation of T-naïve lymphocytes with production of T helper 1 (Th1) and Th2 cytokines (44).

The production of anti-staphylococcus IgE can directly activate the complement factors in the nasal mucosa furtherly feeding the inflammatory process (45). In asthma, S.aureus enterotoxins have been used to phenotype patients and to predict the evolution of comorbidities such as CRSwNP (46).

## 2 Anti-alarmins biologicals drugs currently available: alarmins as an upstream therapeutic target for type 2 diseases

**Figure 1:**
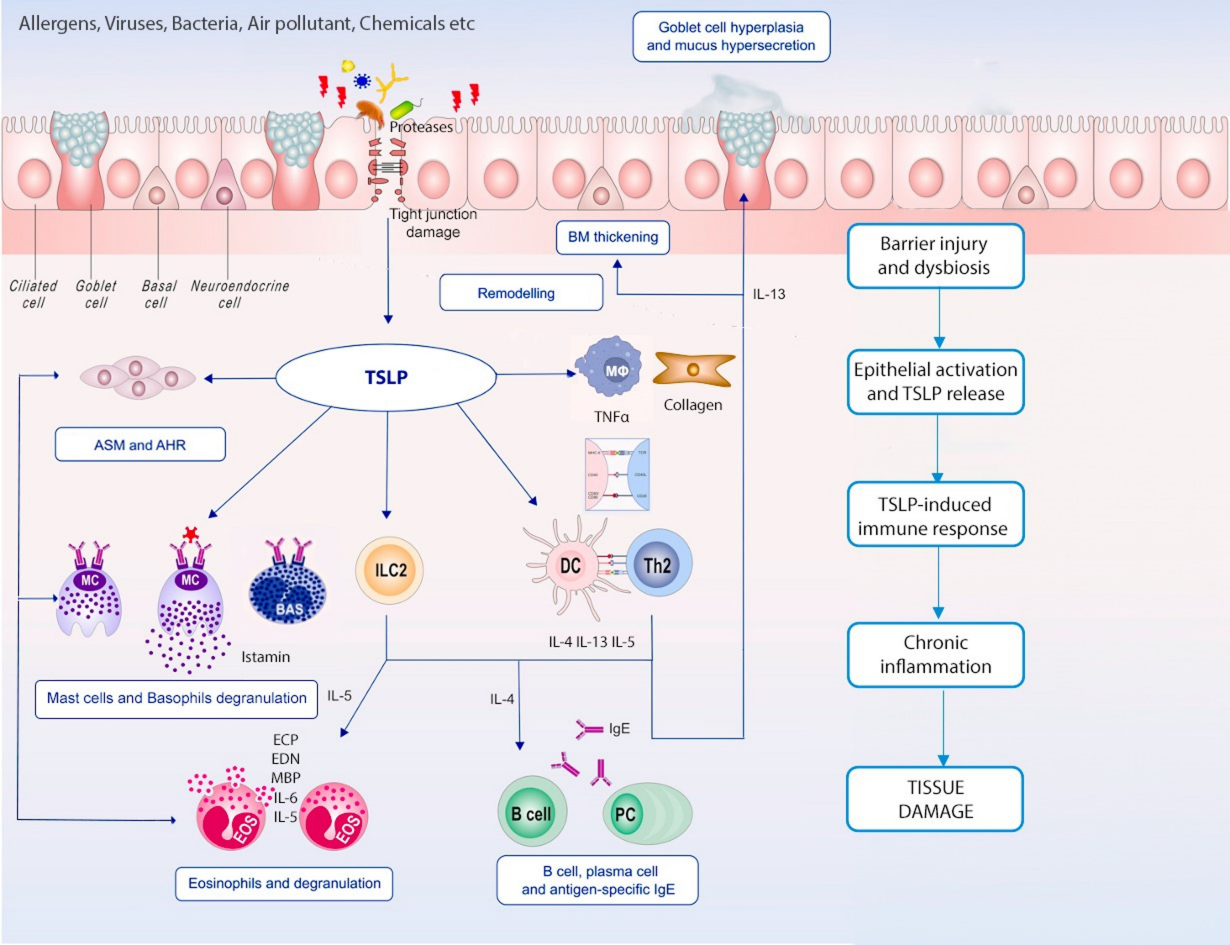
TSLP in chronic inflammation. Epithelial barrier dysfunction and epithelium immune cells’ crosstalk is remarkable in both T2 and non-T2 inflammation; TSLP plays a pleiotropic effect on innate and adaptive immune cells leading to different inflammatory pathways activation, airways remodelling and chronic tissue damage.

Given the complexity of the inflammatory cascades responsible for T2 diseases, treatment outcomes with biological drugs targeting downstream cytokines are not always satisfying. The upstream role that alarmins play makes them a promising novel therapeutic target, as they control many inflammatory patterns and cell types (**Figure 1**). Herein we summarise currently available or in development anti-alarmins biologics.

### 2.1 Tezepelumab: anti-TSLP

Tezepelumab, a fully human IgG2λ, is the first and only approved monoclonal antibody that specifically inhibits TSLP. In the phase 3 NAVIGATOR study, tezepelumab 210 mg every 4 weeks reduced asthma exacerbations independently of biomarkers level over 52 weeks. The most significant effect on exacerbation reduction was detected in patients with allergic eosinophilic asthma (71% in patients with Peripheral Blood Eosinophils (PBE) ≥300 cells/μL and allergen-sensitised; 77% in patients with PBE ≥300/μL and Fractional Exhaled Nitric Oxide (FeNO) ≥25 ppb) and unique results were shown in patients with low T2 biomarker levels (41% reduction of exacerbations in PBE <300 patients and 45% reduction in patients with triple negative signature PBE <150/μl, FeNO<25 ppb, non-allergic). Improvement on lung function, expressed by Forced Expiratory Volume in the first second (FEV1), and asthma control were also clinically significant as well as reduction of biomarkers such as eosinophils, FeNO and total IgE and cytokines IL-4, IL-5 and IL-13. Additional analysis showed that tezepelumab markedly reduced exacerbations precipitated by exposure to specific seasonal and perennial aeroallergens during periods of heightened exposure. This evidence demonstrated that the allergenic trigger could induce epithelial-driven exacerbations that tezepelumab is able to control (47). The phase 3 trial SOURCE showed a non-significant difference between treatment and placebo at 48-weeks on Oral Corticosteroids (OCS)-sparing effect (48). Further studies to reassess tezepelumab efficacy on this outcome are currently ongoing. DESTINATION, the long-term safety and tolerability phase 3 study, with a unique placebo-controlled design, showed the positive benefit-risk profile of tezepelumab over 104 weeks with less adverse event in the treatment arm than in the placebo one (49).

Tezepelumab was approved by FDA as an add-on maintenance treatment for asthma in 2021 as the only available biologic able to suppress all T2 biomarkers and with clinical efficacy not only in T2 asthma but even in non-T2 (50).

As TSLP has been found overexpressed in serum and keratinocytes of patients with AD, activates DCs to produce IL-4, IL-5, IL-13 and TNF-α and contributes to pruritus, it is reasonable to consider as a potential therapeutic target for AD. However, despite numerical results better than placebo, tezepelumab 280 mg every 2 weeks didn’t reach statistical significance in reducing Eczema Area and Severity Index (EASI)-50, EASI-75, EASI-90, SCORing Atopic Dermatitis (SCORAD) and pruritus Numeric Rating Scale (NRS) at 12 weeks in the phase IIa trial ALLEVIAD, on patients with AD (51).

Comorbidities involving both upper and lower airway diseases typically stem from a shared underlying immunologic response initiated by epithelial barrier damage, leading to “unified airway disease” definition, particularly in the context of the T2 immunologic endotype. Regarding CRSwNP, a 52-weeks clinical trial to investigate the effectiveness of tezepelumab is currently ongoing (52). Promising data in severe asthma patients with comorbid CRSwNP are already available: tezepelumab demonstrated clinically meaningful effect on sinonasal symptoms with the greatest improvements seen for the sleep, function and nasal domains of Sino-Nasal Outcome Test (SNOT)-22 questionnaire.

### 2.2 Itepekimab: anti IL-33

Itepekimab is a human IgG4P monoclonal antibody against IL-33. In a phase 2 trial on 296 patients with moderate to severe asthma randomly assigned to itepekimab, dupilumab, itepekimab plus dupilumab or placebo group, itepekimab showed loss of asthma control after discontinuation of Inhaled Corticosteroids/Long Acting Beta2-Agonists (ICS/LABA) in 22% of patients. Besides, dupilumab and itepekimab in monotherapy, but not in combination therapy, increased FEV1 as compared with placebo with mean improvement 0.16 l and 0.14 l respectively. Even if asthma control was better in dupilumab group, itepekimab treatment showed improvement in asthma control and quality of life and reduced PBE, total IgE and FeNO with statistical significance vs placebo. Hypereosinophilia was reported in dupilumab-alone group, but not in combination and itepekimab groups, suggesting that itepekimab may block downstream IL-5 signalling. No significant improvement was seen in asthma control and FEV1 in patients with PBE <300/μl, suggesting itepekimab will not be useful as non-T2 asthma treatment (53).

### 2.3 Brodalumab: anti IL-25

IL-25 (also known as IL-17E) binds to a heterodimeric receptor complex composed of two subunits (IL-17RA and IL-17RB) for signal transduction. Brodalumab is an anti-IL-17RA human monoclonal antibody (IgG2) which prevents IL-17A, IL-25 and IL-17F signalling. In a phase IIa study, brodalumab showed no significant efficacy on Asthma Control Questionnaire (ACQ), lung function and asthma symptoms after 12 weeks of treatment, in patients with inadequately controlled moderate-to-severe asthma (50).

## 3 Discussion

In chronic inflammatory airway diseases different endotypes have been described, with T2 or non T2 immune responses involved in the inflammatory process underlying disease heterogeneity (54–56).

The chronic inflammation characterised by an active eosinophilic infiltrate and T2 cytokines production is prevalent in chronic inflammatory airway diseases and contributes to the physiological changes (57–59).

The epithelial cells produce TSLP as a crucial initiator and regulator of innate and adaptive immune responses (60). As described above, TSLP expression from the airway epithelium induces the recruitment and infiltration of DCs (61), exerts direct effects on mast cells, basophils, eosinophils activation and potently induces IL-4, IL-5 and IL-13 production (56, 62). TSLP promotes eosinophil survival and induces significant production of IL-6, an eosinophil-derived neurotoxin, chemokines, and chemokine ligands. TSLP increases the expression of Intercellular Adhesion Molecules (ICAM)-1 and CD18 and suppresses the expression of surface L-selectin promoting eosinophil transmigration and accumulation in tissues (63) involving the Extracellular signal Regulated Kinase (ERK), p38 Mitogen-Activated Protein Kinase (MAPK), and Nuclear Factor κ light chain enhancer of activated B cells (NF-κB) signalling pathways (64). TSLP can also induce the formation of eosinophil extracellular traps, which play an important role in innate immune responses to infectious agents that significantly contribute to tissue damage in asthmatic airways (63).

In patients with allergic asthma, it was reported a correlation between the level of immunopositive staining for TSLP in bronchial biopsies and airway eosinophilia 24 hours after allergen exposure (65). In contrast, TSLP concentration in asthma patients induced sputum during virus-induced exacerbations was inversely related to the number of eosinophils suggesting that different mechanisms of action of TSLP could occur in acute exacerbations compared with chronic eosinophilic inflammation (66).

The reduction of barrier function strongly contributes to immune system activation (67): a defective epithelial barrier and a dysfunctional repair system of damaged mucosa increase the vulnerability of the sub-epithelial layer lamina propria to pathogens invasion, inducing a local immune response that, in nasal mucosa for instance, contributes to NP formation process (34).

Inflammatory infiltrate contributes to epithelial barrier damage worsening, establishing a vicious circle through secretion of several cationic proteins, including Major Basic Protein (MBP), which induce a marked decrease in the number of desmosomes and an exfoliation of epithelial cells increasing the epithelium permeability. The mucosal permeability allows allergens and other noxious substances to penetrate the barrier (37, 68).

In the last decades, the employment of monoclonal antibodies to treat moderate-to severe asthma, CRSwNP and AD has deeply modified the therapeutic approach.

It is well known that TSLP initiates T2 inflammatory responses and most recent evidence supports TSLP role in non-T2 immune responses as well (7, 56). Eosinophils, basophils, mast cells, Airway Smooth Muscle Cells (ASMCs), ILC2s, lymphocytes, DCs, hematopoietic progenitor cells, and monocytes/macrophages (69) are part of the broad spectrum of cell types that express TSLP receptor where TSLP exert a direct effect between immune and epithelial cells in the airways (7).

As described above, the role of the epithelial barrier dysfunction and epithelium immune cells’ crosstalk is remarkable in both T2 and non-T2 inflammation; different types of organs and tissues share the disruption of the epithelial barrier integrity as a source of continuous triggers for chronic inflammatory damage. By targeting the effector cells, through the blockage of IL-5 or IL-4 and IL-13, T2 inflammation could be well controlled. However, there is emerging evidence about the role of molecules active at the top of the inflammatory cascade that could be exploited as therapeutic targets in a wider range of pathologies thanks to a wider range of immunological effects.

Therefore, TSLP could be considered as a promising therapeutic option in allergic eosinophilic diseases since they are primarily epithelial-driven. Moreover, it could be considered in non-T2 inflammatory diseases, considering its contribution in various inflammatory endotypes.

Given the importance of epithelial barrier damage in promoting different inflammatory diseases, targeting epithelial cytokines could have not only a therapeutic implication but could also have a preventive role, avoiding the amplification of the signals that lead to the chronic inflammatory damage.

There’s a vicious circle of the epithelial barrier damage-inflammatory pathways activation-clinical symptoms development and, its modulation with new treatment options should be considered as a future direction in managing chronic inflammatory diseases.

## Data availability statement

The original contributions presented in the study are included in the article/supplementary material. Further inquiries can be directed to the corresponding author.

## Author contributions

IB: Data curation, Methodology, Supervision, Writing – original draft, Writing – review & editing. SC: Data curation, Methodology, Supervision, Writing – original draft, Writing – review & editing. AL: Data curation, Writing – original draft. AP: Data curation, Supervision, Writing – review & editing. AG: Supervision, Writing – review & editing. CC: Data curation, Methodology, Project administration, Supervision, Writing – original draft, Writing – review & editing.

## Glossary

**Table:**

TSLP	Anti-thymic stromal lymphopoietin
ECs	Epithelial Cells
T2	Type 2
HDM	House Dust Mites
TLRs	Toll-Like Receptors
NOD	Nucleotide-binding Oligomerization Domain
CLRs	C-type Lectin Receptors
RLRs	Retinoic acid-Inducible Gene (RIG)-I-Like Receptors
DCs	Dendritic Cells
Th2	T helper 2
ILC2s	type 2 Innate Lymphoid Cells
Th17	T helper 17
CSE	Cigarette Smoke Extract
TNF	Tumour Necrosis Factor
ASMCs	Airway Smooth Muscle Cells
ROS	Reactive Oxygen Species
AD	Atopic Dermatitis
EoE	Eosinophilic Esophagitis
CRS	Chronic Rhinosinusitis
NP	Nasal Polyps
SNS	Hyperchromatic Supranuclear Stria
CRSwNP	Chronic Rhinosinusitis with Nasal Polyps
Th1	T helper 1
PBE	Peripheral Blood Eosinophils
FeNO	Fractional Exhaled Nitric Oxide
FEV1	Forced Expiratory Volume in the first second
OCS	Oral Corticosteroids
EASI	Eczema Area and Severity Index
SCORAD	SCORing Atopic Dermatitis
NRS	Numeric Rating Scale
SNOT	Sino-Nasal Outcome Test
ICS/LABA	Inhaled Corticosteroids/Long Acting Beta2-Agonists
ACQ	Asthma Control Questionnaire
ICAM	Intercellular Adhesion Molecules-1
ERK	Extracellular signal Regulated Kinase
MAPK	Mitogen-Activated Protein Kinase
NF-κB	Nuclear Factor κ
MBP	Major Basic Protein
